# Enhanced O_2_/N_2_ Separation of Mixed-Matrix Membrane Filled with Pluronic-Compatibilized Cobalt Phthalocyanine Particles

**DOI:** 10.3390/membranes10040075

**Published:** 2020-04-18

**Authors:** S. A. S. C. Samarasinghe, Chong Yang Chuah, H. Enis Karahan, G. S. M. D. P. Sethunga, Tae-Hyun Bae

**Affiliations:** 1 Singapore Membrane Technology Centre, Nanyang Environment and Water Research Institute, Nanyang Technological University, Singapore 637141, Singapore; suechathu@gmail.com (S.A.S.C.S.); chongyang.chuah@ntu.edu.sg (C.Y.C.); hekarahan@ntu.edu.sg (H.E.K.); dilharap001@e.ntu.edu.sg (G.S.M.D.P.S.); 2 Interdisciplinary Graduate School, Nanyang Technological University, Singapore 637335, Singapore; 3 School of Chemical and Biomedical Engineering, Nanyang Technological University, Singapore 637459, Singapore; 4 Department of Chemical and Biomedical Engineering, Korea Advanced Institute of Science and Technology, Daejeon 305-338, Korea

**Keywords:** O_2_/N_2_ separation, Matrimid, cobalt(II) phthalocyanine, pluronic, mixed-matrix membrane

## Abstract

Membrane-based air separation (O_2_/N_2_) is of great importance owing to its energy efficiency as compared to conventional processes. Currently, dense polymeric membranes serve as the main pillar of industrial processes used for the generation of O_2_- and N_2_-enriched gas. However, conventional polymeric membranes often fail to meet the selectivity needs owing to the similarity in the effective diameters of O_2_ and N_2_ gases. Meanwhile, mixed-matrix membranes (MMMs) are convenient to produce high-performance membranes while keeping the advantages of polymeric materials. Here, we propose a novel MMM for O_2_/N_2_ separation, which is composed of Matrimid^®^ 5218 (Matrimid) as the matrix, cobalt(II) phthalocyanine microparticles (CoPCMPs) as the filler, and Pluronic^®^ F-127 (Pluronic) as the compatibilizer. By the incorporation of CoPCMPs to Matrimid, without Pluronic, interfacial defects were formed. Pluronic-treated CoPCMPs, on the other hand, enhanced O_2_ permeability and O_2_/N_2_ selectivity by 64% and 34%, respectively. We explain the enhancement achieved with the increase of both O_2_ diffusivity and O_2_/N_2_ solubility selectivity.

## 1. Introduction

Separation of oxygen (O_2_) and nitrogen (N_2_) from the air has attracted a vast amount of interest in the medical and chemical industries [1]. For instance, the application of oxygen-enriched air is often preferred in fuel combustion processes since an increased oxygen content in the oxidant gas assures a higher energy efficiency in the overall process [2,3]. In addition, oxygen-enriched air can improve treatment efficiency in sewage treatment plants [4]. On the other hand, high-purity nitrogen is used in food preservation to allow a longer storage time, in coal extraction to extinguish potential release of fires during the mining process [5], in the creation of an inert atmosphere in laboratory syntheses and chemical transport [6,7,8], and in cryogenic storage with the use of liquid nitrogen [9,10,11,12].

Conventionally, cryogenic distillation and pressure-swing adsorption have been extensively used in air separation processes. Even though these technologies are able to produce high-purity gases in large quantities, substantial encumbrance in terms of high energy consumption and capital cost [13,14] is foreseen. Thus, recently, membrane-based gas separation has attracted substantial research interest due to its simplicity and cost-effectiveness. Nevertheless, polymeric membranes that are commonly utilized in gas separation processes suffer from the trade-off relationship between permeability and selectivity [15,16] as the gas transport in such membranes is governed by the solution-diffusion mechanism. On the other hand, pure molecular sieve membranes are still hard to be utilized in industrial applications due to their poor scalability [17]. Therefore, the development of a mixed-matrix membrane (MMM), which combines the advantages of both polymeric membranes and molecular sieves, has been considered as a technically-viable option to produce high-performance membranes in a scalable manner [18,19].

At the present stage, effective separation of O_2_ and N_2_ from the air is an uphill struggle due to their close effective diameters (O_2_: 2.89 Å; N_2_: 3.04 Å) [20,21]. The small difference in their diameters brings a great challenge for employing molecular sieving (by porous fillers such as zeolites or reticular frameworks) as the sole driving force of separation. In addition, most adsorbents tend to show favorable adsorption towards N_2_ over O_2_ due to the former possessing higher polarizability (O_2_: 17.4 × 10^−25^ cm^3^; N_2_: 15.8 × 10^−25^ cm^3^) [22]. Nevertheless, certain metal-organic frameworks (MOFs), such as chromium(II) carboxylate MOF (Cr_3_BTC_2_) and MOF-5, possess high O_2_/N_2_ selectivity. However, their structural instability with the increase in the number of operating cycles limits the prospects of practical use [23,24,25]. On the other hand, polymers of intrinsic microporosity (PIMs) have shown high-performance for O_2_/N_2_ separation [26,27,28]. Nonetheless, polymer aging is highly evident in PIMs, leading to a substantial decrease in membrane performance over time [29,30,31,32]. At this point, the facilitated carriers (as molecules and solid particles) offer new opportunities for designing composite membranes for O_2_/N_2_ separation.

Facilitated carriers typically favor the permeation of one of the gas species in a gas mixture by the hopping mechanism. For the case of O_2_/N_2_ separation, the hopping mechanism takes place in the selective and reversible complexation of O_2_ by jumping from one carrier to another through transport events, while N_2_ is inert [33]. Among the carrier alternatives for facilitating the transport of O_2_, cobalt-based complexes, namely cobalt(II) phthalocyanine (CoPC), cobalt(II) tetraphenylporphyrin (CoTPP), and cobalt(III) acetylacetonate (Co(acac)_3_), stand out for their O_2_ selectivity [33,34,35,36,37]. Considering its wide availability and low cost, we decided to test the effect of CoPC on the O_2_/N_2_ separation performance of polyimide-based membranes. Aimed at achieving a high-performance MMM design, we selected Matrimid^®^ 5218 as the polymer matrix, which provides an intrinsic O_2_/N_2_ selectivity close to six [22].

The permeability of Matrimid^®^ 5218 needs improvement for fabricating a high-performance MMM out of it. As observed by Midda and coworkers using the polysulfone-CoPC system [38], non-selective voids forming between the polymer matrix and carriers might enhance gas permeability. Nevertheless, non-selective voids might lead to the formation of more substantial defects, causing a dramatic reduction in selectivity performance as well [39]. Therefore, it is imperative to compatibilize CoPC particles with the polymer matrices very well. To this end, the surface modification of CoPC particles with tert-butyl groups, for example, is a viable strategy [34,38]. However, an additional step for chemical modification is typically necessary. Alternatively, as reported by several groups, block copolymers might serve as efficient compatibilizers between fillers and polymer matrices [40,41]. As such, this research introduces the innovative idea to compatibilize fixed carriers in polymer matrices using suitable block copolymers. The approach to accomplish this task is described in detail in the experimental section.

Here, we report a high-performance MMM for O_2_/N_2_ separation based on Matrimid^®^ 5218 (in short, Matrimid) as a polymer matrix, CoPC microparticles (CoPCMPs) as fixed-site carriers, and Pluronic^®^ F-127 (in short, Pluronic) as a block copolymer compatibilizer. Based on the gas permeation analysis, the incorporation of 5 wt.% CoPCMP and 10 wt.% Pluronic improves the O_2_ permeability and O_2_/N_2_ selectivity by 68% and 34%, respectively. By performing a solubility/diffusivity analysis, we found that the Pluronic-compatibilized Matrimid-CoPCMP system exhibits improvement in both O_2_ diffusivity and O_2_/N_2_ solubility selectivity as compared to the neat polymer (Matrimid). Given each component of the Matrimid-Pluronic-CoPCMP system is commercially available and reasonably affordable, it is justifiable to expect that the demonstrated MMM design is promising for large-scale membrane production.

## 2. Materials and Methods

### 2.1. Materials

**Figure 1:**
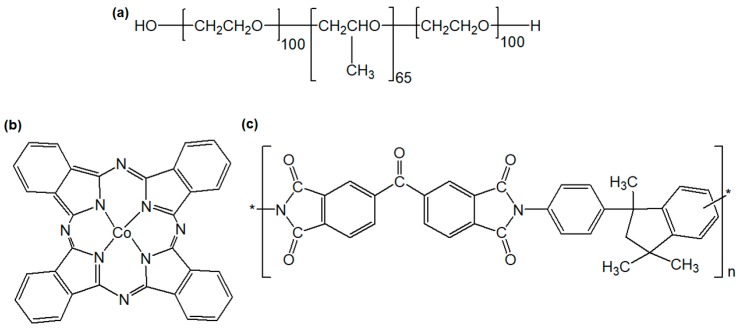
Chemical structures of (**a**) Pluronic^®^, (**b**) cobalt(II) phthalocyanine microparticles (CoPCMPs), and (**c**) Matrimid^®^.

Cobalt(II) phthalocyanine microparticles (CoPCMPs) and Pluronic^®^ F-127 (Pluronic) were purchased from Sigma-Aldrich. *N*,*N*-dimethylformamide (DMF) was purchased from VWR (Radnor, PA, USA). Matrimid^®^ 5218 (Matrimid) was purchased from Huntsman Corporation (Conroe, TX, USA). All chemicals were used as received without further purifications. Chemical structures of CoPCMPs and commercial polymers are illustrated in Figure 1.

### 2.2. Membrane Fabrication

Pure Matrimid membrane was fabricated by dissolving 0.5 g of polymer in 2.3 g of DMF. The dope solutions containing CoPCMPs were prepared by mixing the required amounts of Matrimid, CoPCMPs, and/or Pluronic inside glass vials, which was followed by the addition of DMF, according to the literature, with slight modifications [38,42]. For instance, the composite membrane containing 85 wt.% Matrimid, 5 wt.% CoPCMPs, and 10 wt.% Pluronic was prepared by adding 0.4 g of Matrmid, 0.05 g of CoPCMPs, and 0.05 g of Pluronic into 2.3 g of DMF. The dope solution was agitated overnight with the aid of magnetic stirring. Once the mixtures became well-mixed to the naked eye, the membranes were prepared by casting on a Teflon-made Petri dish in a glove bag that was filled with DMF vapor. After allowing the membrane to be dried at room temperature for approximately 1 h, the Petri dishes were placed in the oven at 60 °C overnight for pre-drying. Lastly, the pre-dried membranes were further dried and annealed in a vacuum oven at ~76 cm Hg at 160 °C for 24 h prior to the gas permeation test.

### 2.3. Characterization

The morphology of the CoPCMPs was studied by field emission scanning electron microscopy (FESEM) on a JSM-7600F (JEOL, Akishima, Tokyo, Japan). For the preparation of microscopy specimens, the CoPCMPs were dispersed in ethanol, and several drops of CoPCMP dispersion were deposited on a freshly cleaned silicon wafer substrate. For observation of the cross-sectional views of the membrane samples, the annealed membranes were fractured in liquid nitrogen, followed by air drying at room temperature, and platinum sputtering. Energy-dispersive X-ray spectroscopy (EDX, JSM-7600F, JEOL, Akishima, Tokyo, Japan) was further supplemented for the best performing membrane (from the gas permeation test) in order to verify the dispersibility of CoPCMPs in the MMM. The porosity properties of CoPCMPs were measured by N_2_ physisorption at 77 K, where a volumetric gas sorption analyzer (NOVATouch LX2, Quantachrome, Boynton Beach, FL, USA) was utilized. The samples were outgassed at 160 °C for 8 h prior to measurement to remove any residual solvents that were present in the sample. Fourier-transform infrared (FTIR) spectra of CoPCMPs, Pluronic, and membranes (neat Matrimid and MMM) were determined using IRPrestige-21 spectrophotometer (Shimadzu Corporation, Kyoto, Japan). The X-ray diffraction (XRD) patterns of CoPcMPs, Pluronic, and membranes were collected at ambient conditions on a Bruker D2 PHASER (Billerica, MA, USA). The thermal behavior of CoPcMPs, Pluronic, and membranes was measured using a thermogravimetric/differential thermal analyzer (TG/DTA, SDT Q600, TA Instruments, New Castle, DE, USA) between 40 to 700 °C. The heating rate of 10 °C min^−1^ was conducted under purging of pure nitrogen, at the flow rate of 100 mL min^−1^. The density of the membrane was measured using an analytical balance (Mettler Toledo, ME204, Columbus, OH, USA), which uses ethanol as the auxiliary liquid. The mechanical properties of the blended (Matrimid-Pluronic) and neat (Matrimid) membranes were tested at room temperature using a tensile meter (Instron 5543, Norwood, MA, USA) that was equipped with 100 N load cell. The reproducibility of the results was conducted by studying at least three different samples for each membrane and reported with standard deviation.

### 2.4. Gas Adsorption Analysis

Pure O_2_ and N_2_ adsorption isotherms of the membranes were measured at 35 °C under the pressure range of 0–5 bar, using a volumetric gas sorption analyzer (iSorb HP1, Quantachrome, Boynton Beach, FL, USA) Due to low adsorption for both O_2_ and N_2_ at the point of interest (0.21 bar for O_2_ and 0.79 bar for N_2_), the amount of gas adsorbed (*q*) was determined from the extrapolation of the O_2_ and N_2_ isotherm for each membrane, considering that the isotherm is considered linear under this measurement range. The solubility of a gas in the membrane, *S* (e.g., O_2_ and N_2_) can be calculated using the following relationship, as described in the literature [6,43]:

(1) $$S=\frac{q\rho }{p}$$

where *q* is the amount of gas adsorbed per mass of membrane, *p* is the pressure, and *ρ* is the density of the membrane. This calculation assumes that there is no competitive adsorption between O_2_ and N_2_ in the membrane [7,37,44]. Gas diffusivity in the membrane, *D*, could then be calculated by dividing permeability with the solubility.

### 2.5. Gas Permeation Test

The gas permeation tests were performed using a constant pressure-variable volume setup (GTR Tec Corporation, Kyoto, Japan). Helium (He, ≥99.9995%) and compressed air (O_2_/N_2_: 21/79, O_2_ ≥ 99.8%, and N_2_ ≥ 99.9995%) were purchased from Air Liquide Singapore Pte Ltd. The membrane was first mounted onto the permeation cell, with the temperature set at 35 °C. The feed pressure was operated at 1 bar. Throughout the analysis, O_2_/N_2_ mixture and He were flown continuously on the upstream and downstream, respectively, by controlling the flow rates with mass flow controllers. When the concentration of O_2_ and N_2_ did not fluctuate, the downstream gas permeating through the membrane was swept periodically by He. The concentrations of O_2_ and N_2_ were determined using gas chromatography attached to the gas permeation setup. The permeability, *P*, can be computed from Equation (2) below, where *q*, *l*, *a*, *p*, and *t* correspond to concentration, membrane thickness, permeation area, pressure, and measurement time (the time taken for the permeate gas to pass through a measuring pipe in the gas chromatography), respectively. To ensure the reproducibility of the gas permeation results, the measurements were repeated at least three times with different samples for each membrane. We took the average of both permeability and selectivity values and reported the standard deviations with error bars.

(2) $$P=\frac{ql}{apt}$$

## 3. Results and Discussion

### 3.1. Characterization of Facilitated Carrier and Compatibilizer

The structural properties of CoPCMPs and Pluronic were first verified using FTIR (Figure S1a). The characteristic peaks of C−H aliphatic stretching, O−H in-plane bending, and C−O stretching of Pluronic can be observed at the frequency of 2850, 1350, and 1100 cm^−1^, respectively. On the other hand, Co−N bond vibration, C−H plane bending, C−N stretching, and C=C ring deformation of CoPCMPs can be identified at the frequencies of 750, 1100, 1450, and 1550 cm^−1^, respectively. The FTIR spectra of CoPCMPs and Pluronic used in this work were comparable to the results reported in the literatures [45,46]. In addition, the characteristic peaks of CoPCMPs and Pluronic were identified in the powder XRD patterns (Figure S1b), in which the peak positions coincide with the results reported in previous works [46,47]. Thermal stabilities of CoPCMPs and Pluronic were determined using TGA analysis (Figure S1c). Both materials demonstrate the thermal decomposition temperature of 620 °C and 380 °C, respectively, indicating that the annealing temperature of 160 °C does not compromise the overall crystallinity of CoPCMPs and stability of Pluronic when these fillers are incorporated in the Matrimid matrix. Furthermore, the DTA analyses reveal another sharp feature in the heat flows at 55 °C (Figure S1d), which corresponds to the melting temperature of Pluronic [48]. N_2_ sorption of CoPCMPs at 77 K (Figure S2) indicates that the particles utilized in this work do not possess any porosity (Type III isotherm). In addition, based on the *t*-plot analysis (which is used to access the microporosity of a porous material) [49,50], it can be concluded that N_2_ molecules are mostly adsorbed on the external surface (Brunauer-Emmett-Teller (BET) surface area, S_BET_ and external surface area, S_ext_ are similar to each other, in Table S1) of the CoPCMP.

### 3.2. Characterization of Neat, Blended, and Composite Membranes

**Figure 2:**
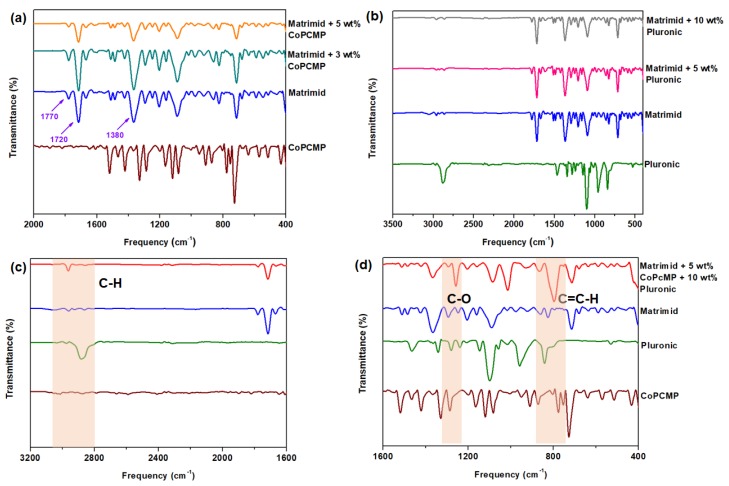
Fourier transform infrared (FTIR) spectra of (**a**) CoPCMP-based membranes and (**b**) Pluronic-based membranes; (**c**,**d**) FTIR spectra of CoPCMP, Pluronic^®^ F-127 (Pluronic), Matrimid^®^ 5218 (Matrimid), and composite (Matrimid-Pluronic-CoPCMP) containing 5 wt.% CoPCMP and 10 wt.% Pluronic. The shift in absorption bands in (**c**,**d**) are indicated for easy comparison.

**Figure 3:**
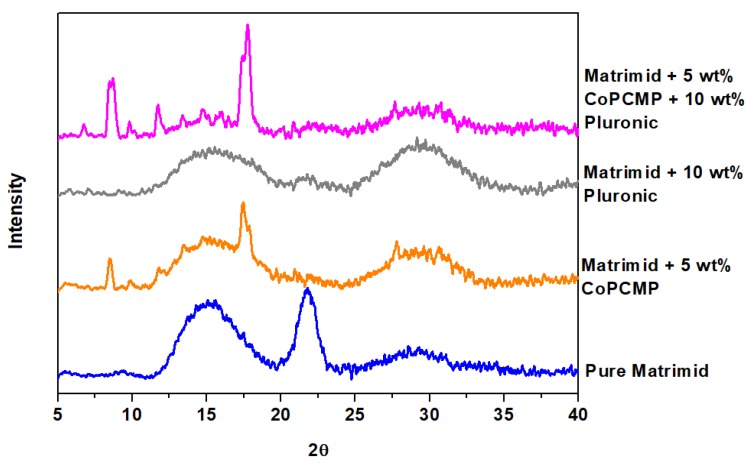
X-ray diffraction (XRD) patterns of neat (Matrimid), blended (Matrimid-Pluronic), and composite (Matrimid-CoPCMP and Matrimid-Pluronic-CoPCMP) membranes.

FTIR measurement was performed on all membranes that were prepared and tested in this work. FTIR spectrum of pure Matrimid (Figure 2a) indicates the presence of characteristic imide feature, with the peaks of 1770, 1720, and 1380 cm^−1^ corresponding to the asymmetric C=O stretching, symmetric C=O stretching, and C–N stretching, respectively, which is in agreement with the results reported in the literature [7]. Superimposition was observed among absorption bands of Pluronic, CoPCMPs, and Matrimid for the case of Matrimid-CoPCMP and Matrimid-Pluronic (Figure 2a,b), where a noticeable shift in their spectra was not observed. Nonetheless, for the case of the Matrimid-CoPCMP-Pluronic system, although the interaction between CoPCMP and Matrimid is weak, the shift of C−H, C−O, and C=C−H bands in Pluronic possibly indicates a potential interaction between Pluronic and Matrimid (Figure 2c,d) [37,51]. In addition, based on the XRD measurement (Figure 3), it was verified that the CoPCMPs remained in crystalline form in the MMM. Pluronic, on the other hand, did not remain in crystalline form upon being blended with Matrimid. Notably, the comparison of TG/DTA profiles of neat/blended and composite membranes indicates that the CoPCMPs did not alter the thermal stability of Matrimid or Pluronic (Figure S3).

**Figure 4:**
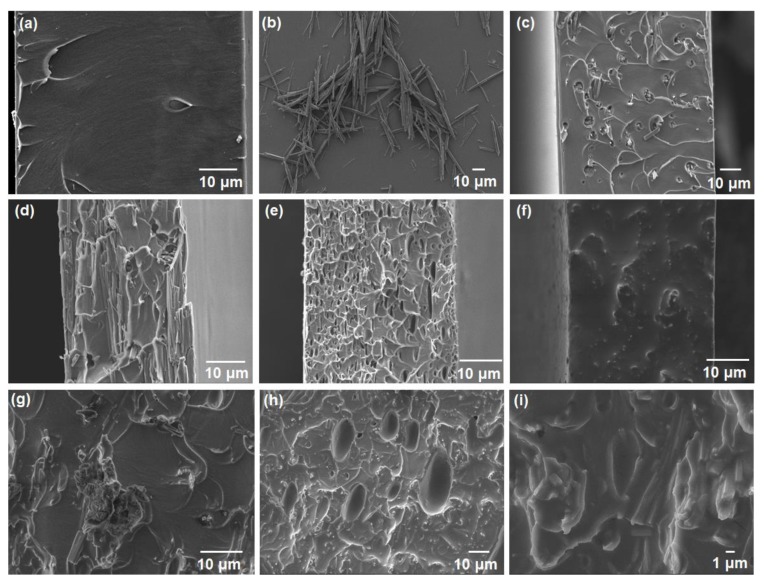
Cross-sectional FESEM images of (**a**) pure Matrimid membrane; (**b**) CoPCMP (deposited on a silicon wafer substrate); (**c**) Matrimid-CoPCMP (3 wt.%); (**d**) Matrimid-CoPCMP (5 wt.%); (**e**) Matrimid-Pluronic (5 wt.%); (**f**) Matrimid-Pluronic (10 wt.%); (**g**) Matrimid-Pluronic-CoPCMP (5 wt.%, 5 wt.%); (**h**) Matrimid-Pluronic-CoPCMP (10 wt.%, 5 wt.%); and (**i**) Matrimid-Pluronic-CoPCMP (10 wt.%, 5 wt.%) at higher magnification.

The interfacial morphologies of the composite membranes provided valuable insights regarding the compatibility of matrices and fillers. Therefore, the cross-sectional views of neat (Matrimid) and blended (Matrimid-Pluronic) membranes were compared with composite membranes (Matrimid-CoPCMP and Matrimid-Pluronic-CoPCMP) under FESEM (Figure 4 and Figure S4). The morphology of CoPCMP is included in Figure 4b. The cross-sectional image of the neat Matrimid (Figure 4a) membrane was considerably smooth, with features attributed to the fracture lines, which appear inevitably. CoPCMPs (Figure 4c,d), on the other hand, caused the formation of a non-ideal interfacial morphology, which was in agreement with previous work [45]. Further increase of CoPCMPs to 10 wt.% (Figure S4a,b) seemingly triggered the aggregation of particles, which can be considered as “defects” in a practical sense. However, the Matrimid-Pluronic blended membrane did not look rich in defects (Figure 4e,f). Such behavior is further supported with the mechanical test of the blended membrane, where an increase in the ductility (decrease in Young’s modulus by 38% for Pluronic-blended membrane) with respect to the neat Matrimid membrane is observed (Table S2). As visual evidence on the promise of the Pluronic-based compatibilization approach, the Matrimid-Pluronic-CoPCMP (10 wt.%, 5 wt.%) gives a much smoother cross-sectional morphology (Figure 4h,i) as compared to Pluronic-free composite membranes. Nonetheless, for the case of Matrimid-Pluronic-CoPCMP (5 wt.%, 5 wt.%), it was observed that the addition of Pluronic compatibilizer was deemed insufficient to heal the “defects” that are present between Matrimid and CoPCMPs. This observation indicates that Pluronic served its desired functionality as a compatibilizer in the Matrimid-Pluronic-CoPCMP system by suppressing the adverse effect of the interfacial incompatibility of Matrimid and CoPCMPs.

### 3.3. Gas Permeation Analysis

The O_2_/N_2_ separation performance of all membranes was evaluated using constant-volume variable pressure gas permeability analysis, and the results are summarized in Table 1. At 3 wt.% CoPCMP, the O_2_/N_2_ selectivity increased by 14.5% at the expense of over 40.7% decrease in O_2_ permeability. When CoPCMP loading increased from 3% to 5 wt.%, the O_2_/N_2_ selectivity further increased from 14.5% to 31.6%, respectively. This is possibly attributed to the barrier effect caused by the agglomeration of CoPCMPs (Figure 4a), as reported in previous work [38]. In contrast, the Matrimid-Pluronic blended membrane exhibited an improved O_2_/N_2_ selectivity at the expense of limited O_2_ permeability. At 10 wt.% loading of Pluronic in the Matrimid membrane, the enhancement of O_2_/N_2_ selectivity was found to be 6.9% with a sharp decrease in O_2_ permeability (55.2%). This is plausibly attributed to the reduction of the fractional free volume of the Matrimid-Pluronic blends due to the potential presence of intermolecular interactions between them, as observed from FTIR spectra (Figure 2c,d), which was also observed in other studies [52,53,54]. On the other hand, the Pluronic-compatibilized MMM (80 wt.% Matrimid, 5 wt.% Pluronic, and 10 wt.% CoPCMP) showed increases in both O_2_ permeability by 64% and O_2_/N_2_ selectivity by 34%, respectively, leading to the enhancement of O_2_/N_2_ separation performance towards a favorable direction (Figure S5). Although their performance is generally inferior (with reference to the upper bound limit), our Matrimid-based membranes offer a higher chance of scalability in comparison to in-house polymers reported in the literature [26,28,55]. These results indicate that the presence of Pluronic at sufficient loading (10 wt.%) helps improve the interfacial morphology between the matrix (Matrimid) and the filler (CoPCMP), which provides performance-based support to the conclusion we reached based on the FESEM image (Figure 4 h,i).

Facilitated carriers such as CoPCMPs generally promote gas separation performance by selectively diffusing one of the gas species (for this case, O_2_) through the hopping mechanism. When the carriers are free to move (as observed in a liquid medium), the transport event is rather straightforward, commonly involving the carrying of the solute as a “cargo.” However, when the carrier is immobilized in a solid matrix (like polymer-based separation membranes), it is of importance to have a homogeneous dispersion of the carriers within the solid matrix for “hopping” events to occur effectively. Thus, it is essential to prevent the aggregation of carriers. The Pluronic-family polymers are commercially available amphiphilic triblock copolymers of hydrophobic polypropylene oxide (PPO) units (as mid-blocks) and hydrophilic polyethylene oxide (PEO) units (as side-blocks) [56]. This amphiphilic nature of Pluronic helps to bridge Matrimid and CoPCMPs, thereby enhancing the compatibility of the resulting composite. As a result, the hopping of the O_2_ molecules takes place in a more homogeneously dispersed web of carrier sites (as supported by EDX mapping of the MMM with 5 wt.% CoPCMP and 10 wt.% Pluronic in Matrimid, Figure S6), which consequently leads to an increase in O_2_ diffusion through the membrane. However, it should be emphasized that the mixing ratio is critical to achieving the desired performance improvement with the Pluronic-based compatibilization approach.

**Figure 5:**
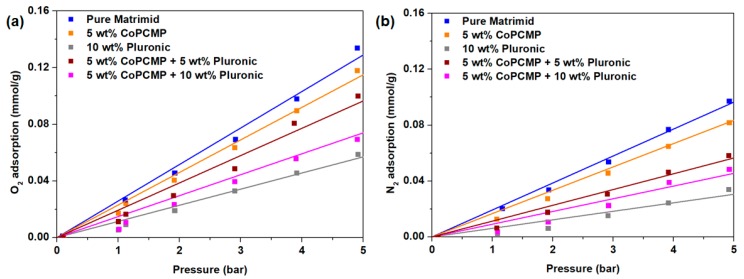
(**a**) O_2_ and (**b**) N_2_ adsorption of neat (Matrimid), blended (Matrimid-Pluronic), and composite (Matrimid-CoPCMP and Matrimid-CoPCMP-Pluronic) membranes at 35 °C at different pressures.

To better explain the nature of performance improvement in our Pluronic-compatibilized composite membrane (Matrimid-Pluronic-CoPCMP), solubility-diffusivity analyses were performed. By measuring the pure component O_2_ and N_2_ adsorption isotherm at 35 °C, the adsorption properties of neat (Matrimid), blended (Matrimid-Pluronic), and composite (Matrimid-CoPCMP and Matrimid-Pluronic-CoPCMP) membranes were first characterized (Figure 5). By doing so and accounting the density of each membrane, a solubility-diffusivity analysis was performed later, as summarized in Table 2. This evaluation, in overall, shows that the incorporation of Pluronic and/or CoPCMP (yielding blended or composite membranes) suppresses the solubility of both O_2_ and N_2_ as compared to the neat Matrimid membrane. Nevertheless, the incorporation of CoPCMPs improves the solubility selectivity, with the enhancement of 11.6% at 5 wt.% loading (Table 2 and Figure S7). On the other hand, the blended membrane (with 10 wt.% Pluronic) suffered from a notable decrease in diffusivity selectivity as compared to the neat Matrimid membrane. However, when all three components are incorporated to form a composite membrane, a harmony between the components takes place, although a sharp decrease in diffusivity selectivity was reported for composite membranes at 50.2% (5 wt.% CoPCMP and 5 wt.% Pluronic) and 40.7% (5 wt.% CoPCMP and 10 wt.% Pluronic) as given in Table 2. Such a strategy allowed us to overcome the limitations of blended (Matrimid-Pluronic) and Matrimid-CoPCMP composite membranes, resulting in significant enhancements in both O_2_ diffusivity and O_2_/N_2_ solubility selectivity.

## 4. Conclusions

Using two commercially available materials, Pluronic and CoPCMP, the O_2_/N_2_ separation performance of Matrimid membrane was successfully enhanced. The CoPCMP served as a functional carrier but failed at performance enhancement without the use of Pluronic, which presumably improved the homogeneity of the resulting membrane. It was found that 5 wt.% CoPCMPs improve both O_2_ permeability (by 64%) and O_2_/N_2_ selectivity (by 34%) when 10 wt.% Pluronic are used for compatibilization, owing to the increases in both O_2_ diffusivity and O_2_/N_2_ solubility selectivity by 12.8% and 37.2%, respectively. Thus, this study exemplifies that no sophisticated strategy is needed to modify carrier particles for achieving performance improvement in O_2_/N_2_ separation. Due to its practicability, the proposed strategy is promising for designing membranes potentially useful for actual applications.

## Figures and Tables

**Table 1:** O_2_/N_2_ gas permeation behavior of neat (Matrimid), blended (Matrimid-Pluronic), and composite (Matrimid-CoPCMP and Matrimid-CoPCMP-Pluronic) membranes at 35 °C under 1 bar (21/79 vol/vol) O_2_/N_2_ feed pressure.

Membrane Composition (wt.%) ^a,b^			O_2_ Permeability (Barrer)	% Change (with Respect to Matrimid)	O_2_/N_2_ Selectivity	% Change (with Respect to Matrimid)
Matrimid	CoPCMP	Pluronic				
100	0	0	1.72 ± 0.29	-	5.79 ± 0.12	-
97	3	0	1.02 ± 0.22	−40.7	6.63 ± 0.08	14.5
95	5	0	1.32 ± 0.32	−23.3	7.62 ± 0.54	31.6
95	0	5	0.93 ± 0.32	−45.9	7.09 ± 1.09	22.5
90	0	10	0.77 ± 0.07	−55.2	6.19 ± 0.64	6.9
90	5	5	1.66 ± 0.15	−3.4	3.82 ± 0.18	−34.0
85	5	10	2.82 ± 0.24	64.0	7.75 ± 1.44	33.9

^a^ The membranes are stated in wt.% in order to show the clarity for each composition; ^b^ Membrane thickness ranges around 60–80 μm, based on the readings made using a micrometer screw gauge.

**Table 2:** Solubility and diffusivity data for neat (Matrimid), blended (Matrimid-Pluronic), and composite (Matrimid-CoPCMP and Matrimid-Pluronic CoPCMP) membranes.

Membrane Composition	Density (g cm^−3^)	O_2_ Solubility (mol m^−3^ bar^−1^)	N_2_ Solubility (mol m^−3^ bar^−1^)	O_2_ Diffusivity (m^2^ s^−1^), ×10^−12^	N_2_ Diffusivity (m^2^ s^−1^), ×10^−12^	O_2_/N_2_ Solubility Selectivity ^a^	O_2_/N_2_ Diffusivity Selectivity ^a^
Matrimid	1.24	31.3	24.3	1.87	0.415	1.29	4.50
5 wt.% CoPCMP	1.25	27.9	19.4	1.60	0.302	1.44	5.30
10 wt.% Pluronic	1.13	12.4	7.0	2.11	0.603	1.77	3.50
5 wt.% CoPCMP, 5 wt.% Pluronic	1.20	23.2	13.6	2.42	1.08	1.70	2.24
5 wt.% CoPCMP, 10 wt.% Pluronic	1.24	20.6	7.1	4.65	1.74	2.90	2.67

^a^ Solubility and diffusivity selectivity is calculated by taking the quotient of O_2_ solubility with N_2_ solubility, as well as O_2_ diffusivity and N_2_ diffusivity, respectively.

## Supplementary Materials

The following are available online at https://www.mdpi.com/2077-0375/10/4/75/s1, Figure S1: Characterization of the facilitated carrier (CoPCMP) and compatibilizer (Pluronic) by (a) FTIR; (b) powder XRD; (c) TGA, and (d) DTA analysis (for the case of Pluronic, due to its onset of degradation occurs around 200 °C; thus the DTA curve beyond 200 °C is indicated as dotted line). Figure S2: N_2_ sorption for CoPCMP at 77 K. Figure S3: (a) TGA and (b) TDA of neat (Matrimid), blended (Matrimid-Pluronic), and composite (Matrimid-CoPCMP and Matrimid-Pluronic-CoPCMP) membranes. Figure S4: Cross-sectional FESEM image of Matrimid-CoPCMP (10 wt.%) under (a) low magnification; (b) high magnification. Figure S5: Comparison of the membrane performance (in Table 1) with the upper bound limit (1991, 2008, 2015). Figure S6: EDX mapping of 5 wt.% CoPCMP and 10 wt.% Pluronic in Matrimid membranes. Figure S7: Solubility and diffusivity selectivity of the studied membranes. Table S1: Porosity properties of CoPCMP. Table S2: Mechanical test of neat (Matrimid) and blended (Matrimid-Pluronic) membranes.
