# Supplementary material for: Enhanced O2/N2 Separation of Mixed-Matrix Membrane Filled with Pluronic-Compatibilized Cobalt Phthalocyanine Particles
Source: Membranes (Basel). 2020 Apr 18;10(4):75. doi: 10.3390/membranes10040075 (PMC7231378; doi:10.3390/membranes10040075)
Supplement: Supplementary file 1 [file membranes-10-00075-s001.pdf]

Supplementary Information for

Enhanced O_2/N_2 Separation of Mixed-matrix Membrane Filled with Pluronic-compatibilized Cobalt Phthalocyanine Particles

S.A.S.C Samarasinghe [1,2,†], Chong Yang Chuah [1,†], H. Enis Karahan [1,3,†],

G.S.M.D.P. Sethunga [1,2], and Tae-Hyun Bae [1,4,*]

[1] Singapore Membrane Technology Centre, Nanyang Environment and Water Research Institute, Nanyang Technological University, Singapore 637141, Singapore

[2] Interdisciplinary Graduate School, Nanyang Technological University, Singapore 637335, Singapore

[3] School of Chemical and Biomedical Engineering, Nanyang Technological University, Singapore 637459, Singapore

[4] Department of Chemical and Biomedical Engineering, Korea Advanced Institute of Science and Technology, Daejeon 305-338, Republic of Korea

* Correspondence: thbae@kaist.ac.kr

[†] These authors contributed equally to this work

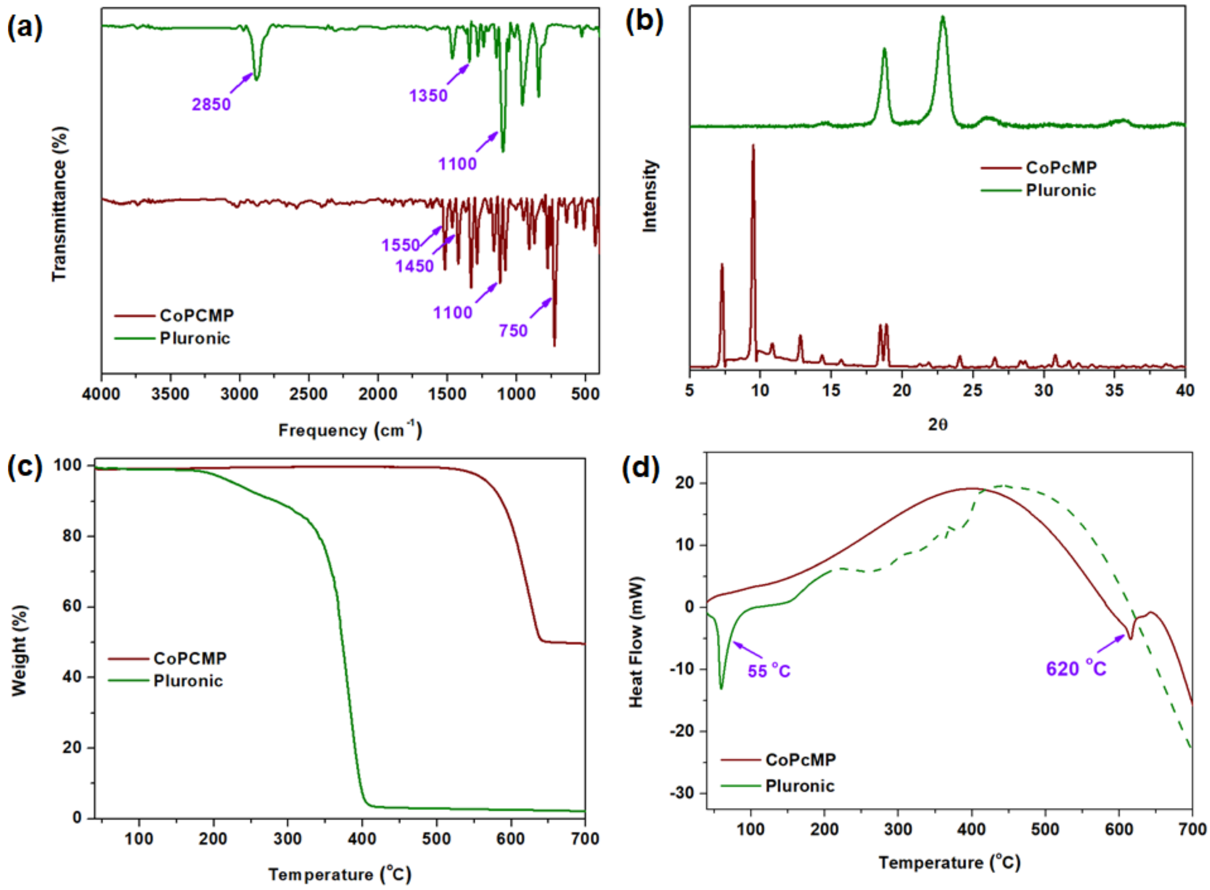

Figure S1. Characterization of the facilitated carrier (CoPCMP) and compatibilizer (Pluronic) by **(a)** FTIR; **(b)** powder XRD; **(c)** TGA and **(d)** DTA analysis (for the case of Pluronic, due to its onset of degradation occurs at around 200 °C; thus the DTA curve beyond 200 °C is indicated as dotted line).

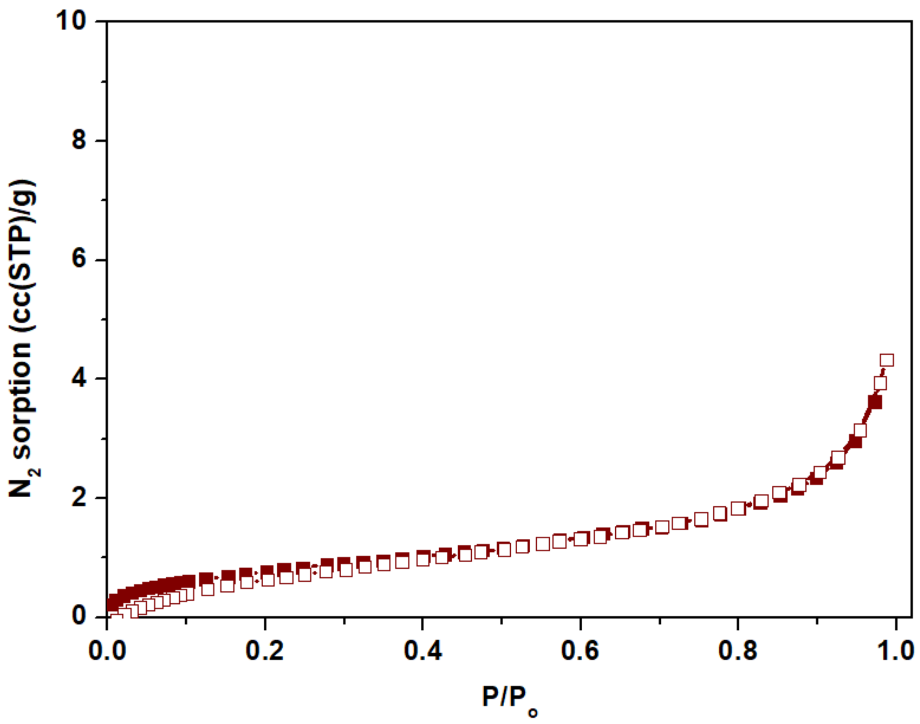

Figure S2. N_2 sorption for CoPCMP at 77 K.

Table S1. Porosity properties of CoPCMP.

Sample	S_{BET}[a] (m²/g)	$S_{Langmuir}$[a] (m²/g)	V_{micro}[b] (cc/g)	S_{ext}[b] (m²/g)	V_{total}[c] (cc/g)
CoPCMP	2.91	4.12	0	2.89	0.0067

[a] S_{BET} and $S_{Langmuir}$ are calculated by selecting P/P_o range from 0.05–0.20.

[b] V_{micro} and S_{ext} are calculated using *t*-plot method, with P/P_o ranging from 0.40–0.60 is selected.

[c] V_{total} is calculated at $P/P_o = 0.99$.

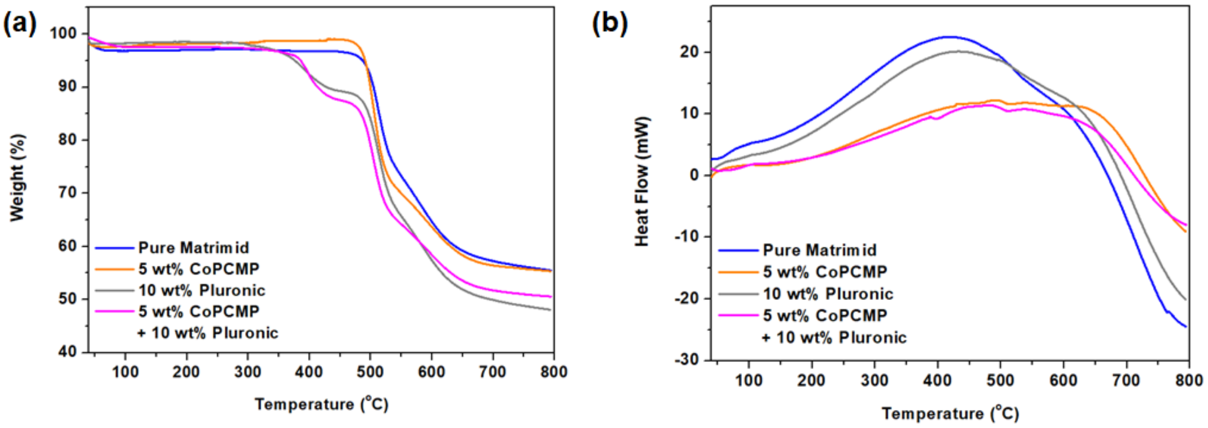

Figure S3. (a) TGA and **(b)** TDA of neat (Matrimid), blended (Matrimid-Pluronic) and composite (Matrimid-CoPCMP and Matrimid-Pluronic-CoPCMP) membranes.

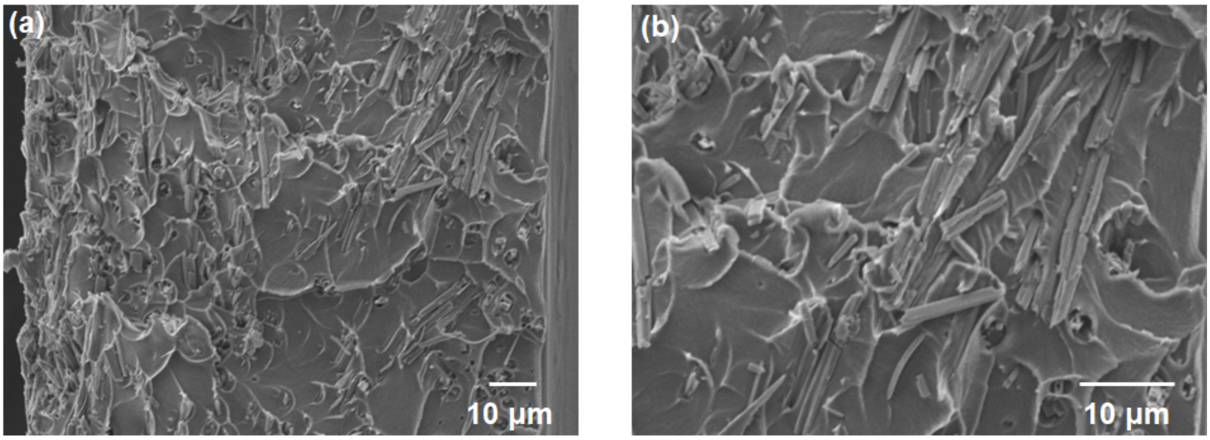

Figure S4. Cross-sectional FESEM image of Matrimid-CoPCMP (10 wt%) under **(a)** low magnification; **(b)** high magnification.

Table S2. Mechanical test of neat (Matrimid) and blended (Matrimid-Pluronic) membranes.

Membranes	Tensile Strength (MPa)	Young Modulus (MPa)
Matrimid	46.6 $\pm$ 2.0	2074 $\pm$ 33
5 wt% Pluronic	65.8 $\pm$ 4.3	1287 $\pm$ 64
10 wt% Pluronic	55.3 $\pm$ 5.9	1286 $\pm$ 97

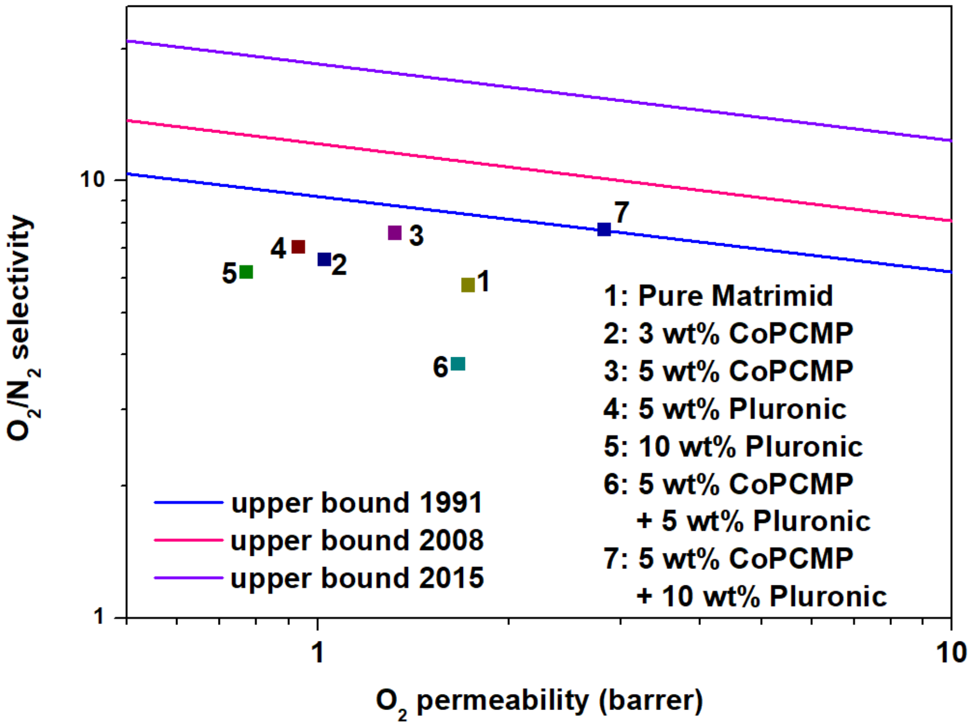

Figure S5. Comparison of the membrane performance (in **Table 1**) with the upper bound limit (1991, 2008, 2015) [1-3].

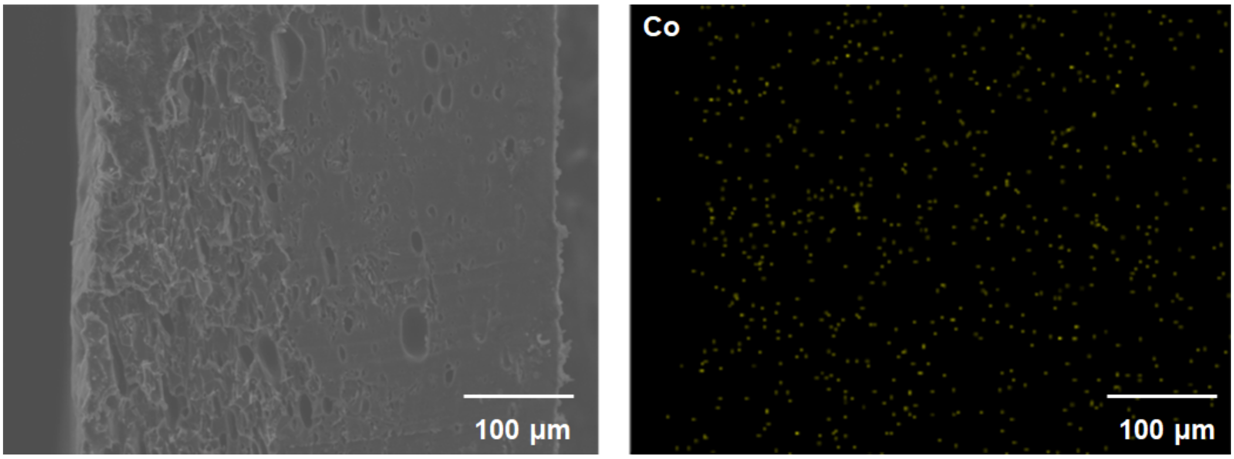

Figure S6. EDX mapping of 5 wt% CoPCMP and 10 wt% Pluronic in Matrimid membranes.

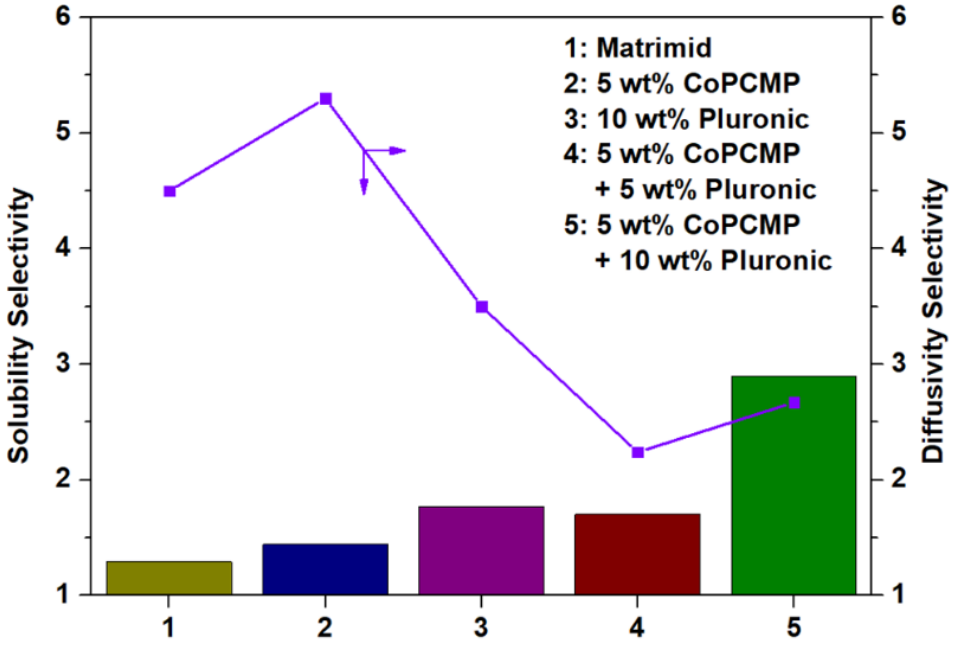

Figure S7. Solubility and diffusivity selectivity of the studied membranes.

References

1. Robeson, L.M. Correlation of separation factor versus permeability for polymeric membranes. *J. Membr. Sci.* **1991**, *62*, 165-185.
2. Robeson, L.M. The upper bound revisited. *J. Membr. Sci.* **2008**, *320*, 390-400.
3. Swaidan, R.; Ghanem, B.; Pinnau, I. Fine-tuned intrinsically ultramicroporous polymers redefine the permeability/selectivity upper bounds of membrane-based air and hydrogen separations. *ACS Macro Lett.* **2015**.
